# Baseline Cerebral Small Vessel Disease Predicting Long‐Term Cognitive Decline in Transient Ischemic Attack Patients

**DOI:** 10.1111/ene.70578

**Published:** 2026-03-26

**Authors:** Paula Roesen, Uchralt Temuulen, Ana Sofia Rios, Ramanan Ganeshan, Tim Bastian Braemswig, Ahmed Khalil, Kersten Villringer, Thomas Ihl, Huma Fatima Ali, Pimrapat Gebert, Ulrike Grittner, Michael Ahmadi, Laurent Puy, Charlotte Cordonnier, Matthias Endres, Heinrich J. Audebert, Anna Kufner

**Affiliations:** ^1^ Klinik für Neurologie mit Experimenteller Neurologie Charité, Universitätsmedizin Berlin, Freie Universität Berlin and Humboldt‐Universität zu Berlin Berlin Germany; ^2^ Charité, Universitätsmedizin Berlin, Freie Universität Berlin and Humboldt‐Universität zu Berlin, Center for Stroke Research Berlin (CSB) Berlin Germany; ^3^ Klinik und Hochschulambulanz für Neurologie Charité Universitätsmedizin Berlin Berlin Germany; ^4^ Berlin Institute of Health at Charité Universitätsmedizin Berlin Berlin Germany; ^5^ Charité, Universitätsmedizin Berlin, Freie Universität Berlin and Humboldt‐Universität zu Berlin, Einstein Center for Neurosciences (ECN) Berlin Germany; ^6^ Charité, Universitätsmedizin Berlin, Freie Universität Berlin and Humboldt‐Universität zu Berlin, Institute of Biometry and Clinical Epidemiology Berlin Germany; ^7^ University Lille, Inserm, CHU Lille, U1172, LilNCog, Lille Neuroscience & Cognition Lille France; ^8^ German Centre for Cardiovascular Research (Deutsches Zentrum für Herz Kreislauferkrankungen, DZHK), Partner Site Berlin Berlin Germany; ^9^ German Center for Neurodegenerative Diseases (Deutsches Zentrum für Neurodegenerative Erkrankungen, DZNE), Partner Site Berlin Berlin Germany; ^10^ German Center for Mental Health (DZPG), Partner Site Berlin Berlin Germany

**Keywords:** cerebral small vessel disease, cerebrovascular risk factors, cognitive impairment, magnetic resonance imaging, transient ischemic attack

## Abstract

**Background:**

Cerebral small vessel disease (CSVD) is a common incidental MRI finding in patients with transient ischemic attack (TIA) and stroke and has been linked to cognitive decline. This study investigated the prevalence of CSVD imaging biomarkers in TIA patients and their association with cognitive performance over 3 years.

**Methods:**

We included 246 TIA patients from the INSPiRE‐TMS study (ClinicalTrials.gov: NCT01586702). CSVD was assessed on baseline 3 T MRI using a composite score (0–4) including white matter hyperintensities (WMH), lacunes, cerebral microbleeds (CMBs), and enlarged perivascular spaces (PVS). Cognitive performance was evaluated using the Montreal Cognitive Assessment (MoCA) at baseline and annually for 3 years.

**Results:**

At least one CSVD imaging biomarker was present in 58.5% of patients. Lacunes (36.6%) were the most common, followed by PVS (28.1%), WMH (19.5%), and CMBs (17.9%). Higher CSVD‐score was independently associated with greater cognitive decline over 3 years (*β* = −0.52, 95% CI −0.95– −0.08, *p* = 0.020), along with older age (*β* = −0.08, 95% CI −0.13 to −0.03, *p* = 0.001). CMB burden was the strongest predictive component of the CSVD‐score (*β* = 0.42, 95% CI −0.63 to −0.22, *p* < 0.001). CSVD‐score was particularly associated with decline in the *memory* domain (adjusted *β* of −0.18, 95% CI −0.32 to −0.04, *p* = 0.015).

**Conclusion:**

CSVD imaging markers are present in over half of TIA patients and are independently associated with cognitive decline up to 3 years, with the strongest effect on *memory*. Whether the presence of CMBs is the strongest predictive imaging biomarker of cognitive decline in TIA patients requires confirmation in further studies.

## Graphical abstract

This study showed that CSVD imaging markers are present in over half of TIA patients and are independently associated with cognitive decline up to 3 years, with the strongest effect on *memory*. Also, CMBs seem to be the strongest predictive imaging biomarker of cognitive decline in TIA patients.

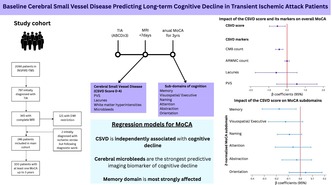

## 1 Introduction

Cognitive decline affects one in five patients globally and represents a significant global health challenge; not only does it negatively affect patient quality of life, but it also poses a substantial socioeconomic burden, especially in an aging global population [1]. Although cognitive decline can have many etiologies—including neurodegenerative diseases, systemic conditions, and vascular pathologies—cerebrovascular disease is one of the leading causes of cognitive impairment in patients over the age of 75 [2]. Cerebrovascular disease not only exacerbates neurodegenerative processes but also independently leads to cognitive deficits by impairing cerebral perfusion and affecting neural networks [3].

Cerebral small vessel disease (CSVD) is often an incidental finding on cerebral magnetic resonance imaging (MRI) in patients following an acute cerebrovascular event [4]. Imaging biomarkers of CSVD include white matter hyperintensities (WMH), lacunes, cerebral microbleeds (CMBs), and enlarged perivascular spaces (PVS) [5]. Recent, large‐scale studies including over 70,000 stroke patients have found that concomitant CSVD is a significant predictor of future cognitive decline [6, 7]. This trend has also been observed in smaller cohorts of transient ischemic attack (TIA) patients [8].

Cognitive impairment after stroke is commonly attributed to ischemia–reperfusion injury, neuroinflammation and strategic infarctions. In these patients WMHs and lacunar strokes often contribute to the cognitive decline and correlate with severity [9]. In contrast, the exact pathophysiology of cognitive impairment after TIA remains less well understood. Minor ischemic lesions (not visible on conventional imaging) and microvascular changes are suspected to play a central role, suggesting that cognitive impairment after TIA is likely related to small vessel disease and underlying microstructural damage that persist after apparently complete clinical recovery [10].

Recent cohort studies including both stroke and TIA patients found that up to 35% of patients experience cognitive decline within 1 year depending on the severity of the initial cerebrovascular event [11, 12]. Previous population‐based studies have shown that CSVD is an independent predictor of cognitive decline, as well as overall long‐term vascular risk and mortality [6, 7]. However, most studies include both ischemic stroke patients and TIA patients, with only a minority (< 30%) representing TIA patients [11, 12]. Although such studies are valuable because ischemic stroke and TIA patients have similar risk profiles, it does not allow for the distinction between the effect of the acute ischemic lesion on cognitive decline and CSVD alone. To the best of our knowledge, there have been no studies as of yet that have investigated the effect of CSVD on long‐term cognitive trajectory in a comprehensive cohort of TIA patients.

Early identification of patients at risk for cognitive decline in groups of TIA patients could lead to early initiation for secondary prevention such as intensified risk factor management, lifestyle interventions, cognitive monitoring and patient education and support. Therefore, we set out to investigate the prevalence of CSVD markers assessed on baseline MRI in a high‐risk TIA population, and whether CSVD is associated with cognitive decline up to 3 years after the acute ischemic event. Furthermore, we investigated whether the severity of CSVD leads to differential decline across cognitive subdomains (i.e., visuospatial/executive abilities, naming, attention, abstraction, memory, and orientation) following TIA.

## 2 Materials and Methods

### 2.1 Participants

This is a post hoc analysis of a subset of data from the INSPiRE‐TMS study (Intensified Secondary Prevention Intending a Reduction of Recurrent Events in TIA and Minor Stroke Patients; ClinicalTrials.gov: NCT01586702). Briefly, INSPiRE‐TMS was an open‐label, multicenter, randomized controlled trial that aimed to explore the effects of an intensified aftercare program in stroke and TIA patients. The main study inclusion criteria were patients with ischemic and hemorrhagic non‐disabling stroke (modified Rankin Scale [mRS] score ≤ 2) or high‐risk TIA (defined as ABCD2 score ≥ 3) [13] within 2 weeks of study enrolment and the presence of at least one modifiable risk factor (i.e., arterial hypertension, diabetes, atrial fibrillation, or smoking). Patients were randomized either to an intensified support programme including up to eight outpatient visits over 2 years including motivational interviewing strategies or to conventional care alone. For a detailed description of the main trial inclusion and exclusion criteria, please refer to Ahmadi et al. 2020 [14].

For the current analysis, patients were included if they had (1) an available MRI (with at least complete T2* or SWI and FLAIR sequences) performed as part of the clinical routine diagnostic workup during the acute hospital stay, (2) the clinical diagnosis of a TIA with (3) no diffusion restriction present on baseline MRI and (4) received assessment of cognitive status on at least one time‐point within 3 years following the index event.

**FIGURE 1:**
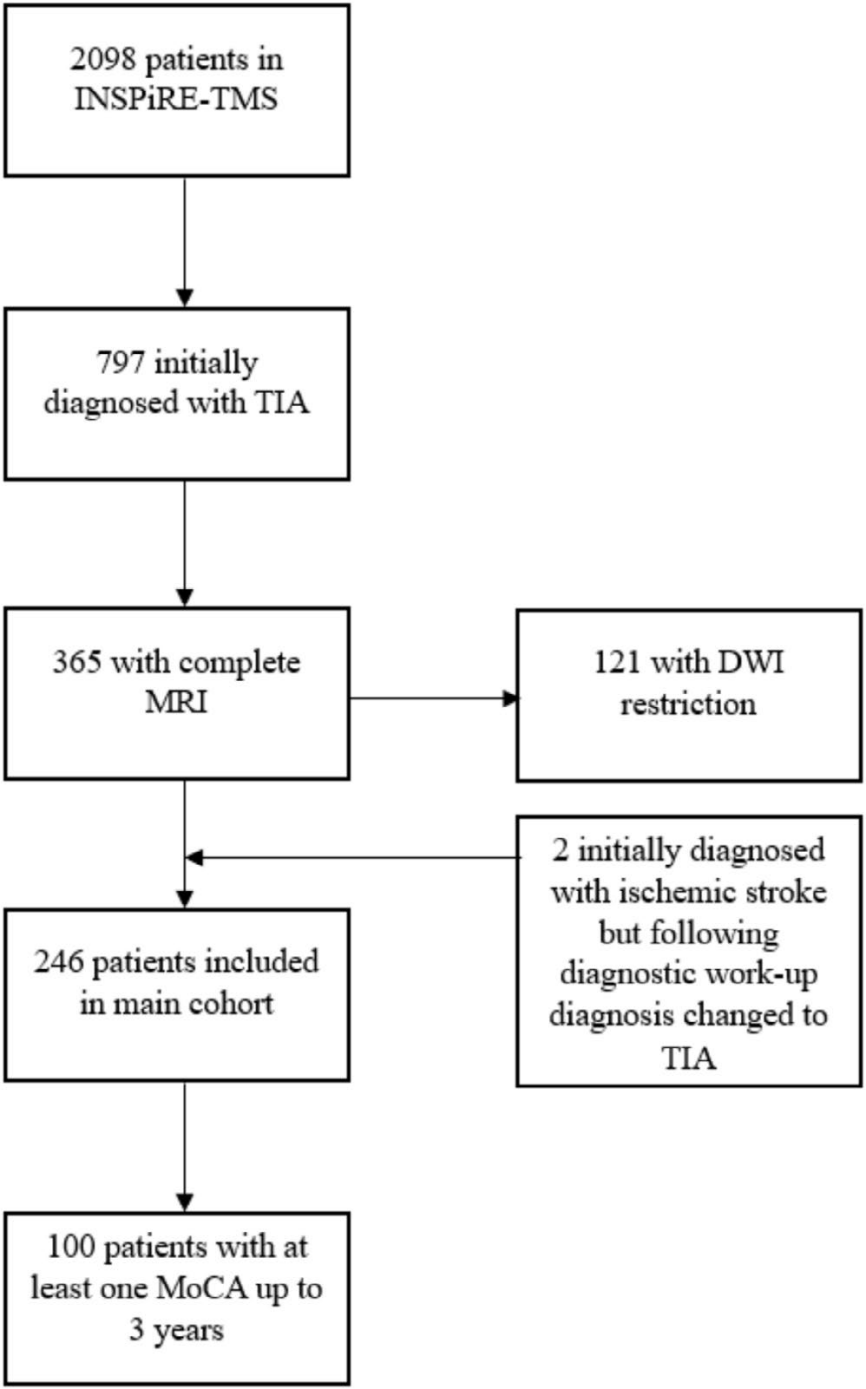
Patient selection flow chart. DWI = diffusion‐weighted imaging, INSPiRE‐TMS = Intensified Secondary Prevention Intending a Reduction of Recurrent Events in TIA and Minor Stroke Patients, MoCA = Montreal Cognitive Assessment, MRI = magnetic resonance imaging, TIA = transient ischemic attack.

Of the 2098 patients enrolled in the INSPiRE‐TMS trial, 797 were initially classified as a TIA based on initial clinical presentation and computed tomography (CT) imaging. Of those, 365 received an MRI and 121 of these showed acute diffusion restriction on the MRI and were subsequently diagnosed with ischemic stroke. Two patients were initially diagnosed with ischemic stroke at the time of study enrolment but were then diagnosed with TIA following further diagnostic work‐up. For a detailed flow‐chart of patients included and excluded in the current study, please refer to Figure 1. In total, 246 patients were included in the final analysis. Out of 246 patients, 100 had at least one Montreal Cognitive Assessments (MoCA) during the 3 years follow‐up. A total of 146 patients dropped out due to missing or not fully completed MoCA.

### 2.2 Outcome Assessment

Cognitive status was assessed using the MoCA score; the score ranges from 0 to 30 in which higher scores represent better cognitive performance [15]. It assesses the subdomains visuospatial abilities/executive functions, naming, attention, abstract thinking, short‐term memory, and orientation to time and place [16]. MoCA was assessed at baseline (during the acute hospital stay), and at annual follow‐up outpatient visits up to 3 years following the acute ischemic event [14]. All analyses involving assessment of cognitive decline were adjusted for age.

### 2.3 Cerebral Small Vessel Disease Score on Baseline MRI

Imaging was performed locally with a 3 Tesla MRI scanner (TimTrio; Siemens AG, Erlangen, Germany). To assess acute diffusion restriction, as well as the CSVD‐score, we used axial diffusion‐weighted imaging (DWI; scanning parameters: TE = 93.1 ms, TR = 7600 ms, FOV = 230 mm, matrix = 192 × 192, 2.5 mm section thickness with no intersection gap), T1‐, T2‐ and T2*‐weighted imaging (scanning parameters: TE = 20.0 ms, TR = 620.0 ms, FOV = 220 mm, matrix = 256 × 192, 5.0 mm section thickness with 0.5 mm intersection gap) and fluid attenuated inversion recovery (FLAIR) sequences (scanning parameters: TE = 100 ms, TR = 8000 ms, FOV = 220 mm, matrix = 256 × 256, 5.0 mm section thickness with 0.5 mm intersection gap).

The MRIs were initially assessed independently by two board certified radiologists. CSVD features were rated independently by two raters (P.R. and H.F.A.) blinded to cognitive outcomes. All ratings were supervised by a physician and imaging expert (A.Ku).

The CSVD‐score is a score ranging from 0 to 4 where points are allocated for the presence of CMBs, lacunes, PVS, as well as relevant white matter hyperintensities (WMH) [17]. Cerebral microbleeds were defined as small areas of signal void with associated blooming on T2* or SWI sequences [18]. The CMB distribution was categorized according to the Microbleed Anatomical Rating Scale [19]. The WMH burden was assessed in FLAIR sequences with the Age‐Related White Matter Changes (ARWMC) score by Wahlund et al. [20] The cutoff was predefined at 10 with ARWMC < 10 as non‐relevant and MRIs with ARWMC ≥ 10 were given a point in the CSVD‐score for significant WMH [21]. The presence of lacunes was assessed on FLAIR, the presence of PVS (rated as relevant if ≥ 10 within the basal ganglia or centrum semiovale) was assessed on T2 or FLAIR sequences using the STRIVE‐2 criteria [18, 22].

### 2.4 Statistical Analysis

To assess factors associated with cognitive decline up to 3 years following the acute cerebrovascular event (TIA), we performed a linear mixed model in which the dependent variable was the MoCA score (continuous) and random intercepts for individuals. In the first linear mixed model, intervention group, age, sex, recurrent stroke (defined as ischemic stroke after enrollment up to 3 years; included as a time dependent variable), time‐point of assessment (baseline, 1‐, 2‐, 3‐year), and total CSVD‐score (range 0–4) were included as fixed effects. Models with and without interaction between time point and total CSVD score were compared using Akaike Information Criterion (AIC) and Bayesian Information Criterion (BIC); the best model was selected based on the lowest AIC and BIC values. In this study, the best model was identified as the model in which no interaction effect between time point and CSVD score was assessed. Normality and homoscedasticity were examined, and we assumed that our models satisfied the key assumptions of the linear mixed model (Figures S1 and S2). For cross‐study comparability, we provided standardized coefficients from the mixed model and the variance‐explained measures (marginal and conditional *R* ^2^) using the package *r2_mlm* [23] in Stata.

In a linear mixed model for MoCA (continuous), the CSVD total score was replaced by individual CSVD component scores, namely CMB count (continuous), presence of enlarged PVS (binary), presence of lacunes (binary), and total WMH burden defined by ARWMH (continuous). To assess potential collinearity among predictors, we ran separate mixed models for individual predictors and compared the results with those from the full model. The consistency of the parameter estimates across these analyses indicated that collinearity was not an issue in our model. For all mixed models, the subjects were included as a random effect (random intercept). Furthermore, we performed a mixed model analysis for new onset MCI (mild cognitive impairment, defined as MoCA< 26) including the following covariates: CSVD‐score, age, timepoint, sex and intervention group as fixed effects [16].

In order to assess whether total CSVD‐score leads to differential decline across cognitive sub‐domains, we z‐normalized the scores within each of the six cognitive sub‐domains including visuospatial/executive, naming, attention, abstraction, memory, and orientation [16]. Subsequently, individual linear mixed models (random intercept models with random intercepts for individuals) were run on each normalized subcategory score used as the dependent variable; fixed effects included intervention group, age, sex, recurrent stroke, time‐point of assessment (baseline, 1‐, 2‐, 3‐year), and total CSVD‐score; subjects were included as a random effect (random intercepts).

A two‐sided significance level of *α* = 0.05 was used. Due to the exploratory nature of the current analysis, no adjustment was made for multiple testing; therefore, *p*‐values should be interpreted with caution. Interpretation of findings is based on effect estimates and corresponding 95% confidence intervals (CI). All statistical analyses were performed using Stata (StataCorp Version 17.0).

## 3 Results

### 3.1 Patient Cohort Description

Of the 246 patients included in the current study, mean age was 69.4 years (standard deviation [SD] 10.4) and 103 patients (41.9%) were female. Median ABCD2 score at baseline was 4 (interquartile range [IQR] 3–5), median National Institutes of Health Stroke Scale (NIHSS) at the time of admission to the stroke unit was 0 (IQR 0–1) and baseline median mRS was 1 (IQR 1–1). No patients had a MoCA of < 26 at baseline. A subgroup of 100 patients had MoCA scores available for at least one time point during the 3‐year follow‐up. Specifically, 98 patients completed the baseline MoCA, 88 completed the 1‐year assessment, 79 completed the 2‐year assessment, and 64 completed the 3‐year assessment. This subgroup had similar patient demographics, with a mean age of 67.1 (SD 10.5) and 43% female. The severity of initial symptoms, based on baseline NIHSS and mRS scores, as well as the distribution of cardiovascular risk factors, was comparable to that of the full cohort. A detailed description of patient characteristics for both the overall cohort and the MoCA‐assessed subgroup is provided in Table 1 and Table S1.

**TABLE 1:** Patient cohort characteristics.

	Total patient cohort (*n* = 246)	Patients with MoCA evaluation (*n* = 100)
*Demographics*		
Age, mean (SD)	69.4 (10.4)	67.1 (10.5)
Sex, female, *n* (%)	103 (41.9)	43 (43.0)
Education level ≥ 10 years, *n* (%)	174 (72.8)	72 (74.2)
*Baseline clinical characteristics*		
ABCD2 Score at admission, Median (IQR)	4 (3–5)	4 (3–4.5)
NIHSS at admission, Median (IQR)	0 (0–1)	0 (0–1)
mRS at admission, Median (IQR)	1 (1–1)	1 (1–1)
TOAST criteria, *n* (%)		
Large‐artery atherosclerosis	15 (6.1)	3 (3.0)
Cardioembolic stroke	39 (15.9)	15 (15.0)
Small‐vessel occlusion	10 (4.1)	3 (3.0)
Other determined etiology	1 (0.4)	1 (1.0)
Undetermined etiology	180 (73.5)	78 (78.0)
*Cardiovascular risk factors*		
Hypertension, *n* (%)	211 (93.7)	88 (91.7)
Diabetes, *n* (%)	57 (25.3)	20 (20.8)
Hypercholesterinemia, *n* (%)	203 (90.6)	87 (90.6)
Atrial fibrillation, *n* (%)	50 (22.4)	19 (20.2)
Current smoking, *n* (%)	25 (10.2)	7 (7)

Abbreviations: IQR = interquartile range, MoCA = Montreal Cognitive Assessment, mRS = modified Rankin Scale, NIHSS = National Institutes of Health Stroke Scale, SD = standard deviation, TOAST = Trial of Org 10,172 in acute stroke treatment.

### 3.2 Prevalence of Cerebral Small Vessel Disease

In the entire cohort (*n* = 246), 58.1% had a CSVD‐score of ≥ 1; 28.5% of the patients had a score of 1, 19.5% a score of 2, 7.7% a score of 3, and 2.9% a score of 4. The most prevalent CSVD marker was the presence of lacunes (36.6%), followed by enlarged PVS (28.1%), WMH (19.5%), and CMBs (17.9%).

The CMB count ranged from 0 to 15 and the most common location was lobar (52.3%), followed by mixed locations with CMB scattered across lobar, deep gray matter, and infratentorial regions (36.4%). The median ARWMC score was 4 (IQR 0–8) and 19.5% of the participants had a high enough score (ARWMC ≥ 10) to be relevant for the CSVD‐score. Full CSVD imaging characteristics of the entire analyzed cohort and subgroup are described in Table 2 and Table S2.

**TABLE 2:** Distribution of cerebral microbleeds, white matter hyperintensities, lacunes, perivascular spaces, and composite CSVD‐Scores in the total cohort (*n* = 246) and MoCA subgroup (*n* = 100).

	Total patient cohort (*n* = 246)	Patients with MoCA evaluation (*n* = 100)
*Cerebral microbleeds*		
Present, *n* (%)	44 (17.9)	17 (17.0)
Count		
1	15 (6.2)	6 (6.0)
2–4	18 (7.3)	6 (6.0)
> 4	11 (4.5)	5 (5.0)
Location		
Deep	0 (0)	0 (0)
Infratentorial	5 (11.4)	3 (17.7)
Lobar	23 (52.3)	5 (29.4)
Mixed	16 (36.4)	9 (52.9)
*White matter hyperintensities*		
ARWMC score, Median (IQR)	4 (0–8)	4 (0–8)
Relevant for CSVD‐score, *n* (%)	48 (19.5)	20 (20.0)
*Lacunes*		
Present, *n* (%)	90 (36.6)	33 (33.0)
*Perivascular spaces*		
Present, *n* (%)	69 (28.1)	21 (21.0)
*Cerebral small vessel disease*		
CSVD‐score, Median (IQR)	1 (0–2)	0 (0–2)
Total CSVD‐score, *n* (%)		
0	102 (41.5)	52 (52.0)
1	70 (28.5)	21 (21.0)
2	48 (19.5)	15 (15.0)
3	19 (7.7)	8 (8.0)
4	7 (2.9)	4 (4.0)

Abbreviations: ARWMC = Age‐Related White Matter Changes, CSVD = cerebral small vessel disease, INSPiRE‐TMS = Intensified Secondary Prevention Intending a Reduction of Recurrent Events in TIA and Minor Stroke Patients, IQR = interquartile range, TIA = transient ischemic attack.

### 3.3 CSVD and Cognitive Trajectory

In the linear mixed model analysis for MoCA scores (treated as a continuous variable) assessed up to 3 years following TIA (*n* = 100), both baseline CSVD‐score (range 0–4) and age were independently associated with cognitive performance. The adjusted *β* for CSVD‐score was −0.52 (95% CI [confidence interval] −0.95 to −0.08, *p* = 0.020), and for age, −0.08 (95% CI −0.13 to −0.03, *p* = 0.001) (Table 3). Timepoint, sex, recurrent stroke and randomization group were not significantly associated with cognitive outcomes in this model (Table 3). The standardized effect size of approximately −0.21 in this model indicates that a 1SD increase in CSVD score was associated with a 0.21SD decrease in MoCA. This represents a small‐to‐moderate effect size relative to the variability in cognitive scores. The fixed effects explained 18.7% of the variance in MoCA scores (marginal *R* ^2^), while the full model including both fixed and random effects explained 68.9% of the variance (conditional *R* ^2^). In an additional mixed model analysis for new onset mild cognitive impairment (MCI; MoCA < 26), CSVD‐score had an adjusted *β* of −0.37 (95% CI −0.79 to 0.03, *p* = 0.071; Table S4). At the 3‐year follow‐up, 42 patients met criteria for MCI based on MoCA scores.

**TABLE 3:** Linear mixed model for MoCA (continuous) assessed up to 3 years following TIA including intervention group, age, sex, time‐point of assessment (baseline as reference) and total CSVD‐score (0–4) as fixed effects (*n*_patients_ = 100, *n*_observertions_ = 329).

Dependent variable: overall MoCA result	Coefficient	95% CI	*p*
CSVD‐score	−0.52	−0.95 to −0.09	0.020
Timepoint			
1 year FU	0.40	−0.09 to 0.88	0.108
2 years FU	0.44	−0.06 to 0.95	0.085
3 years FU	0.35	−0.20 to 0.89	0.210
Age	−0.08	−0.13 to −0.03	0.001
Female sex	0.32	−0.60 to 1.25	0.493
Recurrent stroke	1.05	−0.48 to 2.57	0.177
Randomization group	0.08	−0.84 to 1.00	0.866
*Random effects*			
	Estimate	SE	95% CI
Subject ID	4.39	0.78	3.10–6.21
Marginal *R* ^2^ = 0.19 Conditional *R* ^2^ = 0.69			

Abbreviations: CI = confidence interval, CSVD = cerebral small vessel disease, FU = follow up, MoCA = Montreal Cognitive Assessment, TIA = transient ischemic attack.

In an additional linear mixed model analysis for MoCA (continuous) assessed up to 3 years after TIA, CSVD sub‐components were included as individual fixed effects (Table 4). A significant negative association was found between CMB count and MoCA scores, with an adjusted *β* of −0.42 (95% CI −0.63 to −0.22, *p* < 0.001; Table 4). In this model, age was also negatively associated with MoCA with an adjusted *β* of −0.09 (95% CI −0.13 to −0.04, *p* < 0.001). The other components of the CSVD‐score were not significantly associated with the total MoCA score (Table 4).

**TABLE 4:** Linear mixed model for MoCA (continuous) assessed up to 3 years post TIA including intervention group, age, sex, time‐point of assessment (baseline as reference) and individual CSVD component scores (CMB count, ARWMC score, presence of lacunes and PVS) as fixed effects (*n*_patients_ = 100, *n*_observertions_ = 329).

Dependent variable: overall MoCA result	Coefficient	95% CI	*p*
CMB count (continuous)	−0.42	−0.63 to −0.22	< 0.001
ARWMC score (continuous)	0.00	−0.12 to 0.12	0.958
Lacunes present (binary)	−0.61	−1.75 to 0.53	0.295
PVS present (binary)	0.60	−0.51 to 1.71	0.291
Timepoint			
1 year FU	0.40	−0.09 to 0.88	0.107
2 years FU	0.46	−0.04 to 0.96	0.074
3 years FU	0.34	−0.20 to 0.88	0.214
Age	0.09	−0.13 to −0.04	< 0.001
Female sex	0.58	−0.31 to 1.46	0.201
Recurrent stroke	1.26	−0.21 to 2.72	0.093
Randomization group	0.08	−0.80 to 0.91	0.901
*Random effects*			
	Estimate	SE	95% CI
Subject ID	3.66	0.67	2.56–5.23
Marginal *R* ^2^ = 0.26 Conditional *R* ^2^ = 0.68			

Abbreviations: ARWMC = Age‐Related White Matter Changes, CI = confidence interval, CMB = cerebral microbleed, CSVD = cerebral small vessel disease, FU = follow up, MoCA = Montreal Cognitive Assessment, PVS = perivascular spaces, TIA = transient ischemic attack.

### 3.4 Impact of CSVD‐Score on Cognitive Sub‐Domains

**FIGURE 2:**
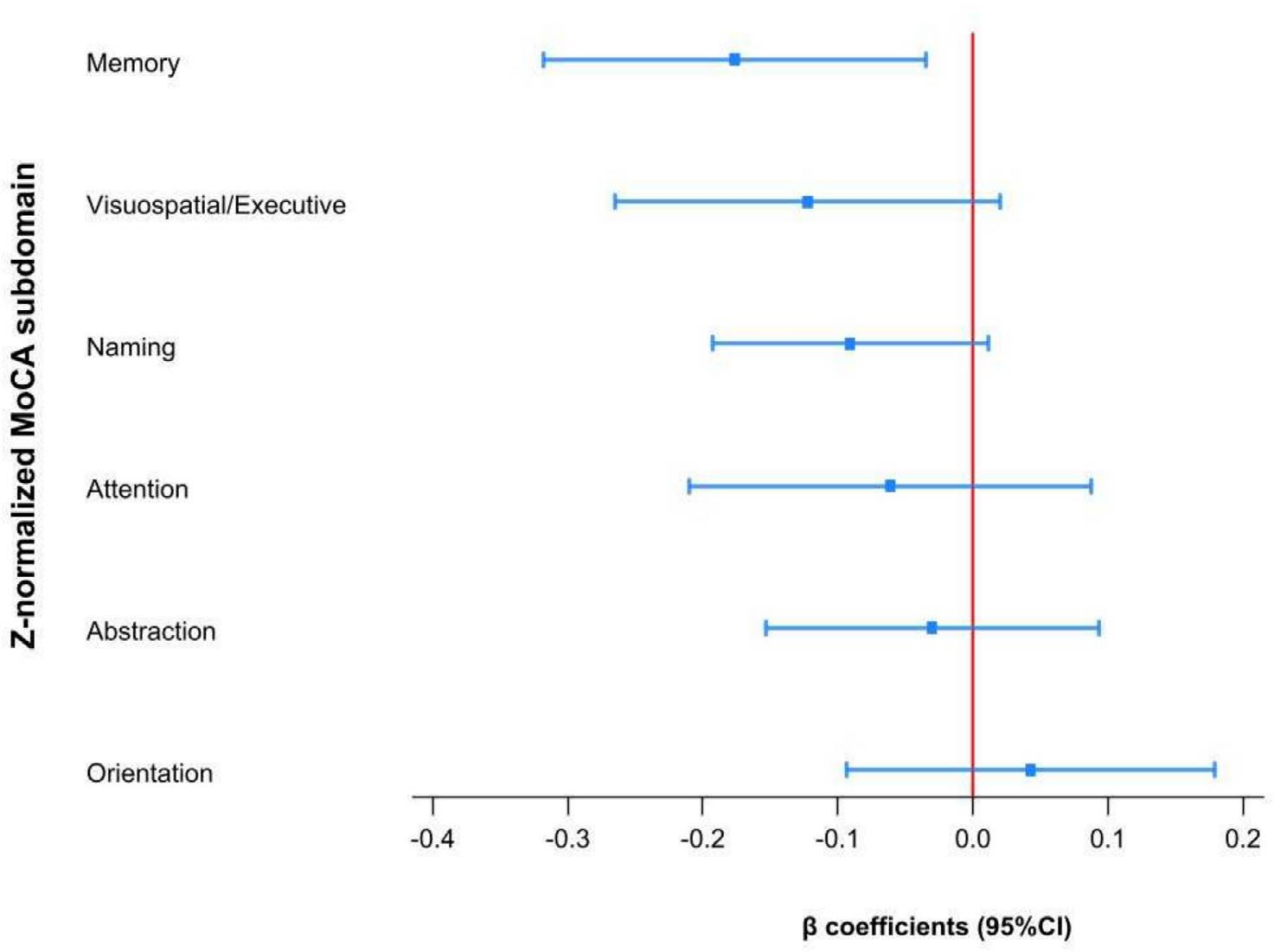
Coefficient plot of impact of CSVD‐score (0–4) on each z‐normalized MoCA subdomain assessed up to 3 years post TIA (*n*_patients_ = 100, *n*_observertions_ = 329); *β* coefficients and 95% confidence intervals of linear mixed model (subcategory score as the dependant variable; fixed effects included intervention group, age, sex, time‐point of assessment and total CSVD‐score; subjects were included as a random effect) depicted. A negative *β* indicates that the more CSVD severity increases (i.e., higher CSVD‐score), the more cognitive performance in the MoCA subdomain worsens (i.e., lower *z*‐normalized score), whereas a positive *β* suggest that higher CSVD severity is associated with better cognitive performance. CI = confidence interval, CSVD = cerebral small vessel disease, MoCA = Montreal Cognitive Assessment, TIA = transient ischemic attack.

For all sub‐domains of cognition except for orientation, higher CSVD‐scores were associated with worse domain‐specific performance (Figure 2), however, the association with the total CSVD‐score was strongest for the *memory* domain (*β* = −0.18, 95% CI −0.32 to −0.04, *p* = 0.015). For the *visuospatial/executive* domain, total CSVD‐score had an adjusted *β* of −0.12 (95% CI −0.26 to 0.02, *p* = 0.103); for *naming*, an adjusted *β* of −0.09 (95% CI −0.19 to 0.01, *p* = 0.088); for *attention*, an adjusted *β* of −0.05 (95% CI −0.21 to 0.09, *p* = 0.476); and for *abstract thinking*, an adjusted *β* of −0.03 (95% CI −0.15 to 0.09, *p* = 0.633). For *orientation*, the CSVD‐score had an adjusted *β* of 0.05 (95% CI −0.09 to 0.18, *p* = 0.5171). Depending on which subdomain, confounding factors such as age and sex had differential effect sizes; the results of each specific mixed model can be found in Table S5.

## 4 Discussion

In this study, we found that not only was CSVD present on MRI in nearly 60% of TIA patients, but that the severity of CSVD—as assessed via the CSVD‐score—was independently associated with worse cognitive outcomes up to 3 years after the acute transient ischemic event, in addition to older age. Although CSVD severity was associated with poorer performance in the specific cognitive domain of *memory*, patients with higher CSVD‐scores were more likely to experience decline in nearly all cognitive subdomains except *orientation*.

This study included 246 moderate‐to‐high‐risk TIA patients (ABCD2 score ≥ 3) from the INSPiRE‐TMS trial, with a mean age of 69 years. In terms of cerebrovascular risk profiles and stroke etiology, the study population is representative of a cardiovascular high‐risk population (Table 1) [24, 25]. More than half of the included TIA patients had a CSVD‐score of ≥ 1, indicating the presence of at least one imaging biomarker of CSVD and incipient cerebrovascular disease (Table 2) [17]. All features of the CSVD‐score were higher in older‐aged patients compared to younger patients (Table S3). These findings align with existing literature that highlights the high prevalence of CSVD markers in populations with vascular risk factors and advanced age [26, 27].

CSVD can present with both occlusive markers—such as lacunar infarcts and WMH, which result from reduced blood flow and subsequent tissue damage—and hemorrhagic markers, including CMBs [28]. In this study, lacunes were the most prevalent imaging marker of CSVD. Previous research has shown that the presence of lacunes not only increases the risk of stroke but may also be associated with greater cognitive impairment [29]. Prominent PVS were the second most frequent MRI feature. Interestingly, previous studies have found that enlarged PVS may be an indicator of microvascular dysfunction and are likely also linked to cognitive decline [22, 30]. A high WMH burden, defined as an ARWMC score ≥ 10, was present in one‐fifth of patients in our study. Previous research has identified WMHs as a well‐established risk factor for both recurrent vascular events and cognitive decline in stroke populations [31, 32]. Hemorrhagic markers of CSVD, specifically CMBs, were the least prevalent imaging feature in this cohort, observed in 18% of TIA patients. CMBs have also been associated with an increased risk of cognitive decline in both aging individuals and stroke survivors [33, 34].

In a linear mixed model analysis, higher total CSVD‐scores were independently associated with poorer cognitive outcomes up to 3 years after the ischemic event (adjusted *β* = −0.52, 95% CI: −0.95 to −0.08, *p* = 0.020), alongside older age (Table 3). These findings are consistent with existing literature [8]. The raw beta coefficient of −0.52 corresponds to an estimated decrease of approximately 0.53 points on the MoCA score for each one‐unit increase in the CSVD marker. To put this in clinical context, the minimal clinically important difference (MCID) for MoCA has been reported to be around 1 to 2 points [35]. In other words, a CSVD score increase from 2 would be considered clinically relevant.

Notably, among the individual CSVD markers, CMBs demonstrated the strongest association with cognitive decline (adjusted *β* = −0.42, 95% CI: −0.63 to −0.21, *p* < 0.001; Table 4). This aligns with evidence from animal models, where induced CMBs led to measurable impairments in cognitive performance [36, 37]. Although several studies have linked CMBs to cognitive dysfunction in mixed stroke and TIA populations [38, 39, 40, 41], high WMH burden has traditionally been viewed as the strongest predictor of cognitive decline [21, 42, 43]. In our cohort, WMH burden was moderate to high (median ARWMC score of 4 [IQR 0–8], with 20% of patients exhibiting a high burden, defined as ARWMC ≥ 10). However, WMH burden alone was not independently associated with cognitive decline in this high‐risk TIA population.

One possible explanation for this discrepancy is that most prior studies focused on stroke cohorts, where WMH burden is higher and likely has stronger prognostic effects. In contrast, among TIA patients, CMBs may serve as a more sensitive indicator of early small vessel pathology [21, 43]. Variations in imaging rating methods and our relatively small sample size could also account for the differing results.

These findings, although needing to be interpreted cautiously because no adjustment for multiple testing was made, highlight the potential importance of CMBs as a marker for identifying TIA patients at increased risk for long‐term cognitive impairment. These results suggest that cognitive dysfunction in TIA patients may stem from the combined burden of CSVD, involving both occlusive and hemorrhagic vascular pathology. Occlusive features (e.g., WMH, lacunar infarcts) arise from chronic vessel narrowing and ischemia, while hemorrhagic features (e.g., CMBs) result from vessel wall fragility and leakage. Both reflect underlying small vessel dysfunction, including impaired vascular reactivity and blood–brain barrier disruption, and can impact different cognitive domains [28].

Patients with more severe CSVD, as indicated by higher total CSVD‐score, performed worse across nearly all cognitive subdomains, with the exception of *orientation*. In this exploratory analysis, total CSVD‐score was significantly associated with poorer *memory* function over the 3‐year follow‐up period (Figure 2). This aligns with the hypothesis that vascular contributions to cognitive impairment disproportionately affect memory and executive functions; possibly due to their reliance on the integrity of widespread neural networks that are particularly vulnerable to ischemic and hemorrhagic injury [3, 44]. Higher CSVD‐scores were also associated with poorer performance in *visuospatial/executive functions, naming, attention*, and *abstract thinking*, suggesting that increased vascular burden may particularly affect these cognitive domains (Figure 2) [36]. In contrast, no substantial association was found with the *orientation* subdomain, likely reflecting its relative resilience in early cognitive decline and the known ceiling effect of MoCA orientation items, which may lack sensitivity to subtle deficits. Ultimately, these findings suggest that progressive CSVD likely causes differential decline in cognitive subdomains depending on overall burden, and the presence of occlusive as well as hemorrhagic imaging markers of CSVD [28]. Future larger, independent studies focusing exclusively on TIA patients are needed to validate these findings and further investigate subdomain‐specific cognitive trajectories in relation to CSVD imaging markers. Given the recognized ceiling effects in the MoCA, our subdomain analyses (e.g., *memory* vs. *attention*) should be interpreted with caution and confirmed through more detailed neuropsychological testing [45, 46].

### 4.1 Limitations

There are several limitations of the current study that warrant discussion. First and foremost, the post hoc and observational design of the study increases the risk of Type II error and limits the ability to establish causality between CSVD and cognitive outcomes. Furthermore, the rather small sample size reduces the capability to detect subtle effects. Because the analyses were exploratory and based on a subset of the INSPiRE‐TMS study, no a priori sample size calculation was possible, and post hoc power analyses are of limited value for model‐based longitudinal analyses. As such, the results should be interpreted with caution and considered hypothesis‐generating rather than confirmatory, and there was also no adjustment made for multiple testing in this exploratory study. Additionally, there is a selection bias, as only patients who received an MRI were included in the current analysis, hereby automatically excluding all patients with contraindications for MRI; patients with MRI contraindications often have more comorbidities, including cardiac pre‐existing conditions [47]. In other words, the generalizability of our findings may be limited. Furthermore, over 30% of the cohort had lacunes—small, chronic ischemic lesions (note: lesion location was not assessed)—which may independently contribute to cognitive impairment, complicating the interpretation of CSVD‐specific effects [44].

Another limitation is the selective inclusion of only high‐risk TIA patients (ABCD2 score ≥ 3), which was intended to ensure diagnostic certainty by capturing clinically probable TIAs. However, this selection bias trade‐off may have come at the cost of generalizability, as it likely excluded patients with lower‐risk profiles or atypical presentations—some of whom may have experienced true TIAs—thus limiting the applicability of our findings to the broader TIA population.

Notably, in this study, CMBs were predominantly located in lobar or mixed regions (Table 2). This distribution raises the possibility that a subset of patients may have had underlying cerebral amyloid angiopathy (CAA), a condition known to present with lobar microbleeds and transient focal neurological episodes (TFNEs) that can mimic TIA. However, CAA was not systematically assessed in this cohort. Therefore, we cannot fully exclude the possibility that some patients had CAA‐related events rather than classical ischemic TIAs. That said, within the MoCA subgroup, only five patients had lobar CMBs, which may limit the potential impact of this confounder on the observed associations with cognitive outcomes. Also, PVS were only rated visually based on their presence and not directly quantified. Although the use of two independent neuroimaging raters partly compensates for this, it does not fully address the limitation of lacking a standardized quantitative assessment.

Additionally, the reliance on MoCA as the primary cognitive assessment tool, while a well‐established and widely used screening tool for cognitive function, may have underestimated deficits in specific domains especially in executive function and processing speed and might have a learning effect when used repeatedly [48, 49, 50]. Larger prospective studies with more in‐depth neuropsychological assessments of cognitive function are needed to validate these findings and address these limitations.

A major strength of this study is the inclusion of TIA patients without acute diffusion restriction on MRI exclusively, reducing the confounding acute lesion effect on cognition [44]. In other words, this design allows for a more precise assessment of the association of CSVD alone on cognitive outcomes. Another strength of the study is the domain‐specific cognitive analysis, combined with its longitudinal design with annual follow‐ups up to 3 years following the index event. In addition to that, this study uses patient data stemming from a large, multicenter randomized controlled trial with comprehensive, longitudinal long‐term follow‐up data. Lastly, all results were reported according to the STROBE criteria (Table S6).

## 5 Conclusion

At the time of a TIA, more than half of patients already show imaging signs of CSVD, which likely influences their long‐term cognitive trajectory. Beyond targeting the cause of the TIA to prevent stroke recurrence, clinicians should consider the presence of CSVD markers—particularly CMBs—as early indicators of cognitive vulnerability. Recognizing and addressing modifiable vascular risk factors associated with CSVD could help shape personalized secondary prevention strategies. If validated in larger, independent cohorts, early MRI‐based assessment of CSVD may become a valuable tool for identifying TIA patients at highest risk of cognitive decline and tailoring interventions accordingly.

## Author Contributions

A.K. and P.R. jointly conceived the study. A.K., P.R., U.G., C.C., L.P., A.R., and P.G. designed and performed data analysis and interpreted the results. P.R. wrote the manuscript. P.R. pre‐processed the MRI data. A.K., U.G., and P.G. gave conceptual and analytical advice. H.F.A. gathered neuroimaging data and helped with image pre‐processing. A.K. performed language assessment and preprocessed the data. U.G. and P.G. helped with statistical analyses. H.J.A. was the Principal Investigator of the INSPiRE‐TMS study. All authors discussed the results and critically revised the manuscript.

## Funding

The INSPiRE‐TMS study was funded within the grant of the German Federal Ministry of Education and Research (BMBF) for the Center for Stroke Research Berlin and co‐funded with unrestricted grants of Pfizer. This project was supported by ERA‐NET NEURON (COHDICH Project, PI: C.C.); Call for Joint Transnational Research Projects 2022; Multinational and translational research projects on Cerebrovascular Diseases including Small Vessel and Brain Barrier Dysfunction and the Bundesminesterium für Forschung, Technologie und Raumfahrt ERA‐NET Neuron 2023‐2026 (01EW2302).

## Conflicts of Interest

M.E. reports grants from Bayer and Ipsen and fees paid to the Charité from Amgen, AstraZeneca, Bayer Healthcare, BMS, Daiichi Sankyo, all outside the submitted work. H.J.A. reports grants from Pfizer and fees paid for lectures and consultancy or data safety boards from AstraZeneca, Bayer Healthcare, Boehringer Ingelheim, BMS, Novo Nordisk, Pfizer, and Roche, all outside the submitted work. L.P. declares the following type of interests: speaker fees (Novonordisk). C.C. reports fees for steering committees from Biogen, Bayer, and for national advisory board from Boehringer‐Ingelheim, all outside the submitted work. She serves as associate editor of the Stroke journal.

## Supporting information


**Figure S1:** Residuals versus fitted plot of linear mixed model.
**Figure S2:** Normal Q‐Q plot of residuals of linear mixed model.
**Table S1:** Patient cohort characteristics in subgroups.
**Table S2:** Cerebral small vessel disease characteristics in subgroups.
**Table S3:** Cerebral small vessel disease markers by age.
**Table S4:** Mixed model for new onset dementia.
**Figure S3:** Coefficient plot of impact of CSVD‐score and its markers on MoCA.
**Table S5:** Cerebral small vessel disease effect on MoCA subdomains.
**Table S6:** STROBE checklist.

## Data Availability

Data supporting the results of this study can be provided upon request.
