# Supplementary material for: Baseline Cerebral Small Vessel Disease Predicting Long‐Term Cognitive Decline in Transient Ischemic Attack Patients
Source: Eur J Neurol. 2026 Mar 26;33(4):e70578. doi: 10.1111/ene.70578 (PMC13140834; doi:10.1111/ene.70578)
Supplement: Supplementary file 1 — Figure S1: Residuals versus fitted plot of linear mixed model. Figure S2: Normal Q‐Q plot of residuals of linear mixed model. Table S1: Patient cohort characteristics in subgroups. Table S2: Cerebral small vessel disease characteristics in subgroups. Table S3: Cerebral small vessel disease markers by age. Table S4: Mixed model for new onset dementia. Figure S3: Coefficient plot of impact of CSVD‐score and its markers on MoCA. Table S5: Cerebral small vessel disease effect on MoCA subdomains. Table S6: STROBE checklist. [file ENE-33-e70578-s001.docx]

**- Supplementary Material –**

**Baseline Cerebral Small Vessel Disease Predicting Long-term Cognitive Decline in Transient Ischemic Attack Patients**

Paula Roesen, Uchralt Temuulen, Ana Sofia Rios, Ramanan Ganeshan, Tim Bastian Brämswig, Ahmed Khalil, Kersten Villringer, Thomas Ihl, Huma Fatima Ali, Pimrapat Gebert, Ulrike Grittner, Michael Ahmadi, Laurent Puy, Charlotte Cordonnier, Matthias Endres, Heiner Audebert, Anna Kufner

**Table of Contents**

**Supplementary Figure 1 (page 2):** Residuals versus fitted plot of linear mixed model

**Supplementary Figure 2 (page 3):** Normal Q-Q plot of residuals of linear mixed model

**Supplementary Table 1 (page 4):** Patient cohort characteristics in subgroups

**Supplementary Table 2 (page 5-6):** Cerebral small vessel disease characteristics in subgroups

**Supplementary Table 3 (page 7):** Cerebral small vessel disease markers by age

**Supplementary Table 4 (page 8):** Mixed model for new onset dementia

**Supplementary Figure 3 (page 9):** Coefficient plot of impact of CSVD-score and its markers on MoCA

**Supplementary Table 5 (page 10-12):** Cerebral small vessel disease effect on MoCA subdomains

**Supplementary Table 6 (page 13-14):** STROBE checklist

**Supplementary Figure 1:** Plot of residuals versus predicted MoCA from the linear mixed model.

**
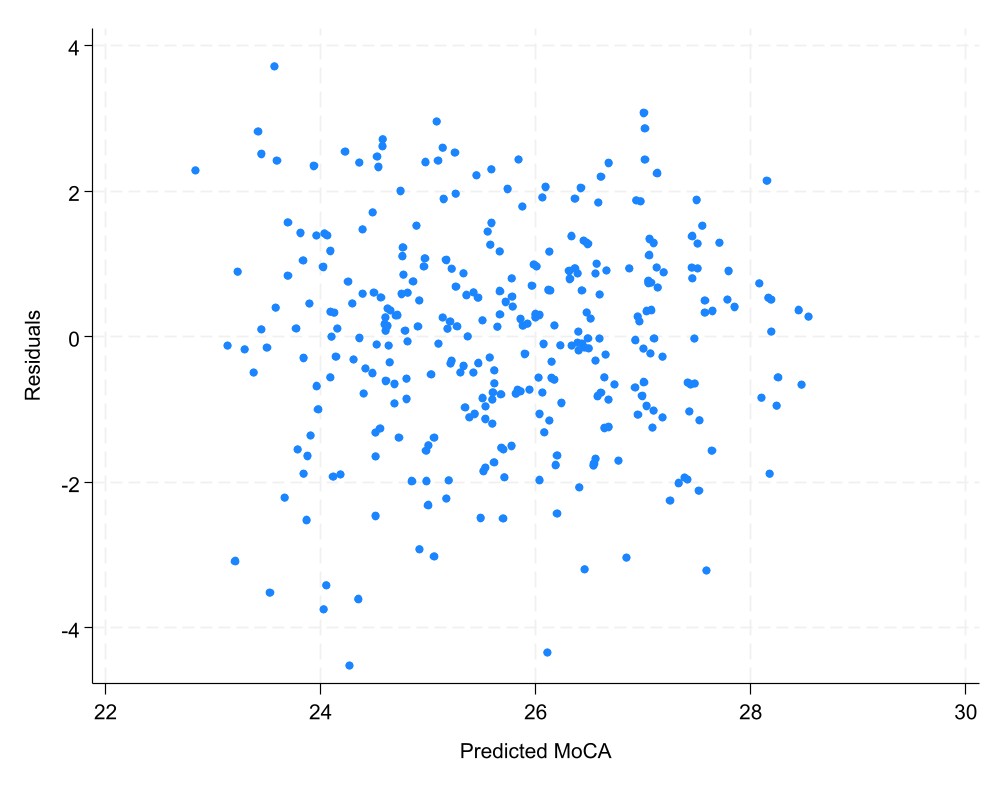
**

MoCA = Montreal Cognitive Assessment

**Supplementary Figure 2:** Normal Q-Q plot of residuals from the linear mixed model predicting MoCA.

**
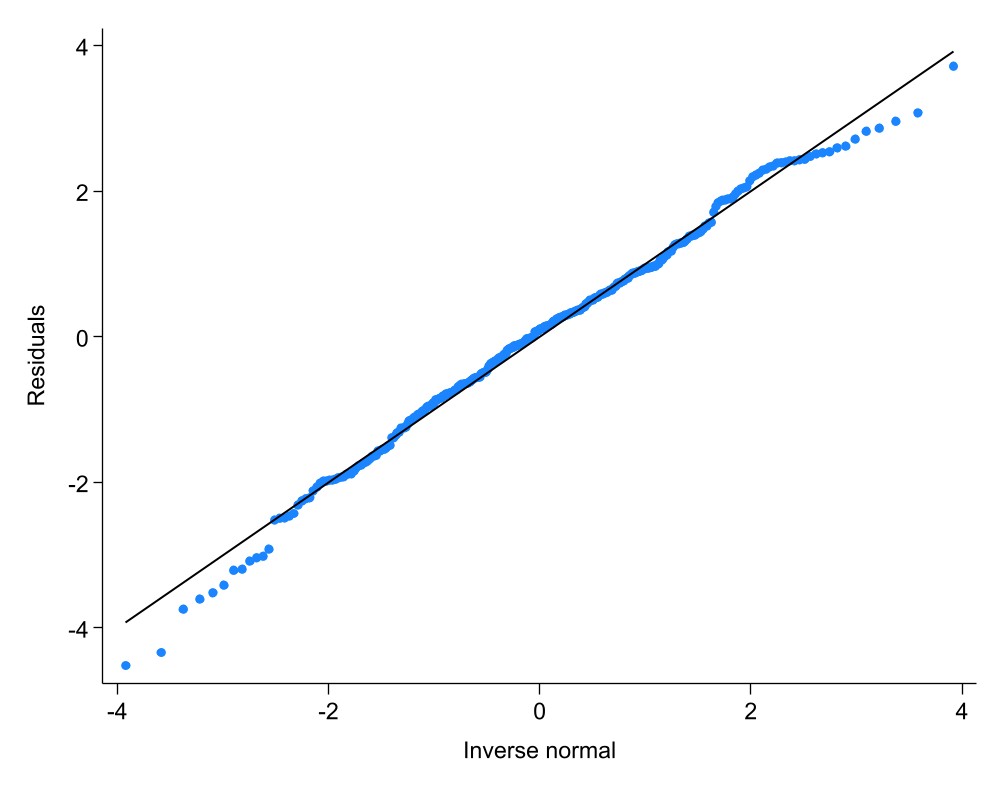
**

MoCA = Montreal Cognitive Assessment

**Supplementary Table 1:** Characteristics of INSPiRE-TMS TIA patient cohort subgroups.

|  | Patients with MoCA evaluation (n=100) | Patients without MoCA evaluation (n=146) |
| --- | --- | --- |
| **Demographics** |  |  |
| Age, mean (SD) | 67.1 (10.5) | 70.8 (10.1) |
| Sex, female, n (%) | 43 (43.0) | 61 (41.8) |
| Education level ≥ 10 years, n (%) | 72 (74.2) | 103 (72.5) |
| **Baseline clinical characteristics** |  |  |
| ABCD2 Score at admission, Median (IQR) | 4 (3-4.5) | 4 (3-5) |
| NIHSS at admission, Median (IQR) | 0 (0-1) | 0 (0-1) |
| mRS at admission, Median (IQR]) | 1 (1-1) | 1 (1-1) |
| TOAST criteria, n (%)  Large-artery atherosclerosis  Cardioembolic stroke  Small-vessel occlusion  Other determined etiology  Undetermined etiology | 3 (3.0)  15 (15.0)  3 (3.0)  1 (1.0)  78 (78.0) | 11 (7.6)  24 (16.6)  7 (4.8)  0 (0)  103 (71.0) |
| **Cardiovascular risk factors** |  |  |
| Hypertension, n (%) | 88 (91.7) | 123 (95.4) |
| Diabetes, n (%) | 20 (20.8) | 37 (28.7) |
| Hypercholesterinemia, n (%) | 87 (90.6) | 116 (90.6) |
| Atrial fibrillation, n (%) | 19 (20.2) | 31 (24.0) |
| Current smoking, n (%) | 7 (7) | 18 (12.3) |
| INSPiRE-TMS = Intensified Secondary Prevention Intending a Reduction of Recurrent Events in TIA and Minor Stroke Patients, TIA = transient ischemic attack, MoCA = Montreal Cognitive Assessment, SD = standard deviation, IQR = interquartile range, NIHSS = National Institutes of Health Stroke Scale, mRS = modified Rankin Scale, TOAST = Trial of Org 10172 in acute stroke treatment | | |

**Supplementary Table 2:** Breakdown of CSVD marker distribution and CSVD severity in INSPiRE-TMS TIA patient cohort subgroups.

|  | MoCA subgroup (n=100) | Non-MoCA subgroup (n=146) |
| --- | --- | --- |
| **Cerebral microbleeds** |  |  |
| Present, n (%) | 17 (17.0) | 27 (19.2) |
| Count:  1  2-4  > 4 | 6 (6.0)  6 (6.0)  5 (5.0) | 9 (6.2)  12 (8,2)  6 (4.8) |
| Location:  Deep  Infratentorial  Lobar  Mixed | 0 (0)  3 (17.7)  5 (29.4)  9 (52.9) | 0 (0)  2 (7.0)  18 (64.3)  7 (28.6) |
| **White matter hyperintensities** |  |  |
| ARWMC score, Median (IQR) | 4 (0-8) | 5 (4-8) |
| Relevant for CSVD-score, n (%) | 20 (20.0) | 28 (19.2) |
| **Lacunes** |  |  |
| Present, n (%) | 33 (33.0) | 57 (39.0) |
| **Perivascular spaces** |  |  |
| Present, n (%) | 21 (21.0) | 47 (32.2) |
| **Cerebral small vessel disease** |  |  |
| CSVD-score, Median (IQR) | 0 (0-2) | 1 (0-2) |
| Total CSVD-score, n (%)  0  1  2  3  4 | 52 (52.0)  21 (21.0)  15 (15.0)  8 (8.0)  4 (4.0) | 50 (34.3)  49 (33.6)  33 (22.6)  11 (7.5)  3 (2.1) |
| CSVD = cerebral small vessel disease, IQR = interquartile range, INSPiRE-TMS = Intensified Secondary Prevention Intending a Reduction of Recurrent Events in TIA and Minor Stroke Patients, TIA = transient ischemic attack, ARWMC = Age-Related White Matter Changes | | |

**Supplementary Table 3:** CSVD in 246 TIA patients broken down by age group.

|  | CSVD-score, mean (SD) | CMB count, mean (SD) | Wahlund score, mean (SD) | Lacunes, n (%) | PVS, n (%) |
| --- | --- | --- | --- | --- | --- |
| Age <60 | 0.20 (0.41) | 0 (0) | 1.28 (2.57) | 5 (12.50) | 2 (5.00) |
| Age 60-80 | 1.12 (1.11) | 0.90 (2.36) | 5.55 (4.48) | 70 (40.70) | 51 (29.65) |
| Age >80 | 1.5 (1.02) | 0.24 (0.74) | 8.29 (5.21) | 15 (44.12) | 16 (47.06) |
| CSVD = cerebral small vessel disease, TIA = transient ischemic attack, SD = standard deviation, CMB = cerebral microbleed, PVS = perivascular spaces | | | | | |

**Supplementary Table 4:** Linear mixed model for new onset MCI (defined as MoCA <26) assessed up to 3 years following TIA including intervention group, age, sex, time-point of assessment and total CSVD-score (0-4) as fixed effects (n_patients_ = 100, n_observations_ = 329).

| **Dependent variable: new onset MCI** | **Coefficient** | **95 % CI** | **p-value** |
| --- | --- | --- | --- |
| CSVD-score | -0.38 | -0.79 – 0.03 | 0.071 |
| Timepoint |  |  |  |
| 1 year FU | 0.94 | 0.18 – 1.70 | 0.016 |
| 2 years FU | 0.31 | -0.45 – 1.07 | 0.429 |
| 3 years FU | 0.84 | 0.00 – 1.67 | 0.050 |
| Age | -0.08 | -0.13 – -0.03 | 0.002 |
| Female sex | 0.43 | -0.45 – -1.31 | 0.338 |
| Randomization group | -0.28 | -1.16 – 0.60 | 0.528 |
| **Random effects** |  |  |  |
|  | **Estimate** | **Std. Error** | **95 % CI** |
| Subject ID | 2.44 | 0.98 | 1.12 – 5.36 |
| MCI = mild cognitive impairment, MoCA = Montreal Cognitive Assessment, TIA = transient ischemic attack, CSVD = cerebral small vessel disease, FU = follow up, CI = confidence interval | | | |

**Supplementary Figure 3:** Coefficient plot of impact of CSVD-score and its markers (CMBs, WMH/ ARWMC score, lacunes, PVS) on overall MoCA assessed up to 3 years post TIA (n_patients_ = 100, n_observations_ = 329); β coefficients and 95% confidence intervals of linear mixed models depicted. A negative β indicates that the more CSVD severity increases (i.e., higher CSVD-score or CMB count), the more cognitive performance worsens (i.e., lower MoCA score). Conversely, a positive β suggest that the presence of i.e. PVS is associated with better cognitive performance.


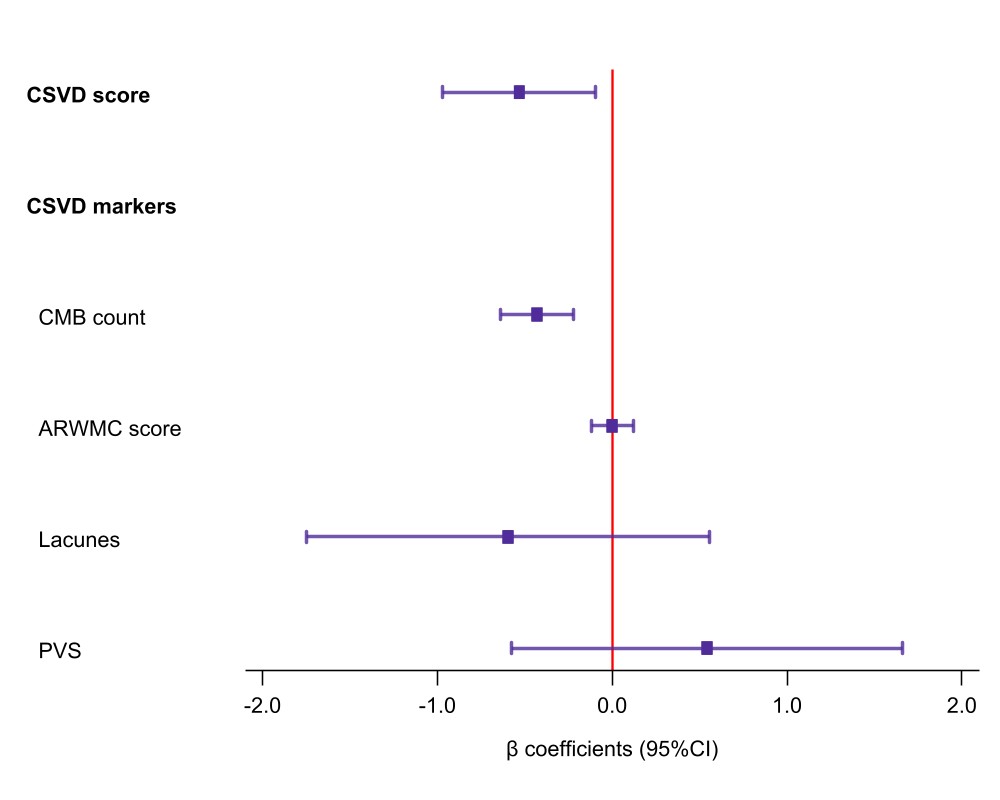


CSVD = cerebral small vessel disease, CMB = cerebral microbleeds, WMH = white matter hyperintensities, ARWMC = Age-Related White Matter Changes, PVS = perivascular spaces, MoCA = Montreal Cognitive Assessment, TIA = transient ischemic attack, CI = confidence interval

**Supplementary Table 5:** Linear mixed models for z-normalized MoCA subdomains (visuospatial/ executive, naming, attention, abstraction, memory and orientation; continuous) assessed up to 3 years post TIA including intervention group, age, sex, time-point of assessment and total CSVD-score (0-4) as fixed effects (n_patients_ = 100, n_observations_ = 329).

|  | **Coefficient** | **95 % CI** | **p-value** |
| --- | --- | --- | --- |
| **Dependent variable: Visuospatial/ Executive** |  |  |  |
| CSVD-score | -0.12 | -0.26 – 0.02 | 0.103 |
| Timepoint |  |  |  |
| 1 year FU | 0.12 | -0.07 – 0.31 | 0.204 |
| 2 years FU | -0.06 | -0.25 – 0.13 | 0.547 |
| 3 years FU | -0.20 | -0.41 – 0.01 | 0.060 |
| Age | -0.03 | -0.04 – -0.01 | < 0.001 |
| Female sex | -0.41 | -0.71 – -0.10 | 0.009 |
| Recurrent stroke | 0.31 | -0.22 – 0.85 | 0.247 |
| Randomization group | -0.10 | -0.40 – 0.21 | 0.530 |
|  |  |  |  |
| **Dependent variable: Naming** |  |  |  |
| CSVD-score | -0.09 | -0.19 – 0.01 | 0.088 |
| Timepoint |  |  |  |
| 1 year FU | 0.00 | -0.28 – 0.28 | 0.986 |
| 2 years FU | -0.10 | -0.38 – 0.19 | 0.514 |
| 3 years FU | -0.27 | -0.58 – 0.04 | 0.086 |
| Age | 0.00 | -0.01 – 0.01 | 0.703 |
| Female sex | -0.16 | -0.37 – 0.05 | 0.141 |
| Recurrent stroke | 0.12 | -0.32 – 0.57 | 0.585 |
| Randomization group | 0.00 | -0.21 – 0.22 | 0.985 |
|  |  |  |  |
| **Dependent variable: Attention** |  |  |  |
| CSVD-score | -0.05 | -0.21 – 0.09 | 0.476 |
| Timepoint |  |  |  |
| 1 year FU | 0.15 | -0.06 – 0.37 | 0.156 |
| 2 years FU | 0.24 | 0.02 – 0.44 | 0.036 |
| 3 years FU | 0.21 | -0.03 – 0.44 | 0.087 |
| Age | -0.01 | -0.02 – 0.01 | 0.347 |
| Female sex | 0.24 | -0.07 – 0.54 | 0.127 |
| Recurrent stroke | 0.61 | 0.05 – 1.17 | 0.034 |
| Randomization group | 0.12 | -0.19 – 0.43 | 0.447 |
|  |  |  |  |
| **Dependent variable: Abstraction** |  |  |  |
| CSVD-score | -0.03 | -0.15 – 0.09 | 0.633 |
| Timepoint |  |  |  |
| 1 year FU | 0.03 | -0.24 – 0-29 | 0.838 |
| 2 years FU | -0.15 | -0.42 – 0.12 | 0.286 |
| 3 years FU | 0.02 | -0.27 – 0.31 | 0.902 |
| Age | -0.01 | -0.03 – 0.00 | 0.060 |
| Female sex | -0.16 | -0.42 – 0.10 | 0.226 |
| Recurrent stroke | 0.01 | -0.51 – 0.53 | 0.964 |
| Randomization group | 0.01 | -0.25 – 0.27 | 0.958 |
|  |  |  |  |
| **Dependent variable: Memory** |  |  |  |
| CSVD-score | -0.18 | -0.32 – -0.04 | 0.015 |
| Timepoint |  |  |  |
| 1 year FU | 0.04 | -0.15 – 0.23 | 0.677 |
| 2 years FU | 0.17 | -0.04 – 0.37 | 0.106 |
| 3 years FU | 0.23 | 0.02 – 0.45 | 0.033 |
| Age | -0.02 | -0.04 – -0.01 | 0.008 |
| Female sex | 0.35 | 0.04 – 0.65 | 0.024 |
| Recurrent stroke | 0.06 | -0.47 – 0.60 | 0.815 |
| Randomization group | -0.07 | 0.37 – 0.23 | 0.646 |
|  |  |  |  |
| **Dependent variable: Orientation** |  |  |  |
| CSVD-score | 0.05 | -0.09 – 0.18 | 0.517 |
| Timepoint |  |  |  |
| 1 year FU | 0.11 | -0.94 – 0.32 | 0.285 |
| 2 years FU | 0.16 | -0.06 – 0.38 | 0.146 |
| 3 years FU | 0.07 | -0.16 – 0.30 | 0.561 |
| Age | -0.01 | -0.03 – 0.00 | 0.074 |
| Female sex | 0.02 | -0.27 – 0.31 | 0.910 |
| Recurrent stroke | 0.18 | -0.35 – 0.72 | 0.508 |
| Randomization group | 0.26 | -0.03 – 0.55 | 0.077 |

MoCA = Montreal Cognitive Assessment, TIA = transient ischemic attack, CSVD = cerebral small vessel disease, FU = follow up

**Supplementary Table 6:** STROBE Statement - checklist of items that should be included in reports of observational studies (with page references)

|  | **Item No.** | **Recommendation** | | **Page  No.** |
| --- | --- | --- | --- | --- |
| **Title and abstract** | 1 | (*a*) Indicate the study’s design with a commonly used term in the title or the abstract | | 1 |
|  |  | (*b*) Provide in the abstract an informative and balanced summary of what was done and what was found | | 3 |
| **Introduction** | | |  | |
| Background/rationale | 2 | Explain the scientific background and rationale for the investigation being reported | | 4 |
| Objectives | 3 | State specific objectives, including any prespecified hypotheses | | 4 |
| **Methods** | | |  | |
| Study design | 4 | Present key elements of study design early in the paper | | 5 |
| Setting | 5 | Describe the setting, locations, and relevant dates, including periods of recruitment, exposure, follow-up, and data collection | | 5 |
| Participants | 6 | (*a*) *Cohort study*—Give the eligibility criteria, and the sources and methods of selection of participants. Describe methods of follow-up  *Case-control study*—Give the eligibility criteria, and the sources and methods of case ascertainment and control selection. Give the rationale for the choice of cases and controls  *Cross-sectional study*—Give the eligibility criteria, and the sources and methods of selection of participants | | 5 |
|  |  | (*b*) *Cohort study*—For matched studies, give matching criteria and number of exposed and unexposed  *Case-control study*—For matched studies, give matching criteria and the number of controls per case | |  |
| Variables | 7 | Clearly define all outcomes, exposures, predictors, potential confounders, and effect modifiers. Give diagnostic criteria, if applicable | | 6-7 |
| Data sources/ measurement | 8* | For each variable of interest, give sources of data and details of methods of assessment (measurement). Describe comparability of assessment methods if there is more than one group | | 6-7 |
| Bias | 9 | Describe any efforts to address potential sources of bias | | 8 |
| Study size | 10 | Explain how the study size was arrived at | | 5 |
| Quantitative variables | 11 | Explain how quantitative variables were handled in the analyses. If applicable, describe which groupings were chosen and why | | 6-7 |
| Statistical methods | 12 | (*a*) Describe all statistical methods, including those used to control for confounding | | 7 |
|  |  | (*b*) Describe any methods used to examine subgroups and interactions | | 8 |
|  |  | (*c*) Explain how missing data were addressed | | - |
|  |  | (*d*) *Cohort study*—If applicable, explain how loss to follow-up was addressed  *Case-control study*—If applicable, explain how matching of cases and controls was addressed  *Cross-sectional study*—If applicable, describe analytical methods taking account of sampling strategy | | 5 |
|  |  | (*e*) Describe any sensitivity analyses | | - |
| **Results** |  |  | |  |
| Participants | 13* | (a) Report numbers of individuals at each stage of study—e.g. numbers potentially eligible, examined for eligibility, confirmed eligible, included in the study, completing follow-up, and analysed | | 5 |
|  |  | (b) Give reasons for non-participation at each stage | | 5 |
|  |  | (c) Consider use of a flow diagram | | 6 |
| Descriptive data | 14* | (a) Give characteristics of study participants (e.g. demographic, clinical, social) and information on exposures and potential confounders | | 9-10 |
|  |  | (b) Indicate number of participants with missing data for each variable of interest | | 9-10 |
|  |  | (c) *Cohort study*—Summarise follow-up time (e.g., average and total amount) | | - |
| Outcome data | 15* | *Cohort study*—Report numbers of outcome events or summary measures over time | | 10-11 |
|  |  | *Case-control study—*Report numbers in each exposure category, or summary measures of exposure | |  |
|  |  | *Cross-sectional study—*Report numbers of outcome events or summary measures | |  |
| Main results | 16 | (*a*) Give unadjusted estimates and, if applicable, confounder-adjusted estimates and their precision (e.g., 95% confidence interval). Make clear which confounders were adjusted for and why they were included | | 11-14 |
|  |  | (*b*) Report category boundaries when continuous variables were categorized | | - |
|  |  | (*c*) If relevant, consider translating estimates of relative risk into absolute risk for a meaningful time period | | - |
| Other analyses | 17 | Report other analyses done—e.g. analyses of subgroups and interactions, and sensitivity analyses | | 12-14 |
| **Discussion** |  |  | |  |
| Key results | 18 | Summarise key results with reference to study objectives | | 15, 18 |
| Limitations | 19 | Discuss limitations of the study, taking into account sources of potential bias or imprecision. Discuss both direction and magnitude of any potential bias | | 17 |
| Interpretation | 20 | Give a cautious overall interpretation of results considering objectives, limitations, multiplicity of analyses, results from similar studies, and other relevant evidence | | 15-17 |
| Generalisability | 21 | Discuss the generalisability (external validity) of the study results | | 17-18 |
| **Other information** |  |  | |  |
| Funding | 22 | Give the source of funding and the role of the funders for the present study and, if applicable, for the original study on which the present article is based | | 19 |

*Give information separately for cases and controls in case-control studies and, if applicable, for exposed and unexposed groups in cohort and cross-sectional studies.
